# The Interplay Between Immunological and Inflammatory Markers as Key Prognostic Indicators in Elderly Patients with COVID-19

**DOI:** 10.3390/healthcare13192477

**Published:** 2025-09-29

**Authors:** Corina Popazu, Violeta Diana Oprea, Alina-Maria Lescai, Aurelia Romila, Marius Petrea, Robert Marius Grosu, Adriana Liliana Vlad, Daniela-Ioanina Prisăcaru, Alexia Anastasia Ștefania Baltă

**Affiliations:** 1 Clinical-Medical Department, Faculty of Medicine and Pharmacy, “Dunarea de Jos” University, Str. Domnească 35, 800201 Galati, Romaniamarius.petrea92@yahoo.com (M.P.); 2 Medical Department, Faculty of Medicine and Pharmacy, “Dunarea de Jos” University, Str. Domnească 35, 800201 Galati, Romania; diana.v.oprea@gmail.com (V.D.O.); aurelia.romila@yahoo.com (A.R.); adriana.vlad.mg3.4@gmail.com (A.L.V.); alexiabalta@yahoo.ro (A.A.Ș.B.); 3 Department of Morphological and Functional Sciences, Faculty of Medicine and Pharmacy, “Dunarea de Jos” University, Str. Domnească 35, 800201 Galati, Romania; grosurobert21@gmail.com; 4 Tübinger Akademie für Verhaltenstherapie, David-von-Stein-Weg 26, 72072 Tübingen, Germany; ioaninaprisacaru@gmail.com

**Keywords:** COVID-19, elderly, inflammatory biomarkers, immune markers, complete blood counts, predictive value

## Abstract

**Background:** The COVID-19 pandemic has disproportionately affected the elderly population, with inflammation and impaired immune response being key drivers of disease progression. Clinicians require predictive models integrating immunological and inflammatory biomarkers to optimize risk stratification in this vulnerable group. **Methods**: We retrospectively analyzed 1429 elderly patients (aged >60 years) admitted with COVID-19 between March 2020 and August 2022. Demographic, clinical, and laboratory data were collected at admission. Correlation and regression analyses were performed to assess the prognostic significance of hematological and inflammatory markers. **Results**: Lymphopenia and neutrophilia were predominant findings, frequently associated with elevated C-reactive protein levels. Correlation analyses revealed significant associations between inflammatory markers and discharge status or death, while lymphocytes exerted a protective effect, reducing mortality risk by 14.4%. Notably, a higher platelet-large cell ratio (PLCR) was linked to increased mortality, suggesting an important contribution of thrombosis to severe COVID-19. **Conclusions**: Our findings indicate that immunological and inflammatory markers may serve as significant predictors of outcomes in elderly COVID-19 patients. While the predictive power of the model remains limited, these biomarkers can contribute to a better understanding of patient trajectories and may inform therapeutic strategies.

## 1. Introduction

The COVID-19 pandemic has had a disproportionate impact on the elderly population, who are considered among the most vulnerable to experiencing severe complications and increased risk of mortality. In particular, immunological and inflammatory markers have attracted attention as possible predictors of clinical evolution. Recent studies have emphasized that inflammation and compromised immune response are key factors in the progression of Coronavirus disease 2019 (COVID-19), especially in elderly patients with a higher incidence of comorbidities and a weaker immune system [1]. However, the precise combinations of immunological and inflammatory markers that could accurately predict the progression of elderly patients remain under-explored.

Recently published research emphasized various potentially predictive biomarkers that could be useful in the early management of the viral disease. Routine, repeated clinical laboratory analysis—such as complete blood counts (CBCs), common acute phase reactants, and targeted cytokine assays—reveal the inflammatory environment. Unsurprisingly, disturbances in numerous CBC parameters are present in the early response to severe acute respiratory syndrome coronavirus-2 (SARS-CoV-2) infection, many of which were found to be predictors of pulmonary disease severity. For example, elevated leukocytosis is almost universally associated with poor outcomes; lymphopenia is also a powerful predictor of severity, development of ARDS, need for ICU care, and death in infected individuals [2]. COVID-19 has been highly associated with alterations in other hemogram parameters, including thrombocytopenia, anemia, and red blood cell distribution width (RDW) [3], but to our knowledge, there are limited data specific for older populations. There are data showing that commonly tested soluble factors have been identified as valuable biomarkers of COVID-19 status and progression [2,3].

Regarding immunology markers, since neutrophilia and lymphopenia are associated with poor outcomes in COVID-19 and other infections, the neutrophil-to-lymphocyte ratio (NLR) has been used to gauge the magnitude of the immune response [3]. Identifying circulating factors downstream of the immune system may prove beneficial in predicting progression and targeting therapies, as demonstrated by an in-depth analysis [4]. Furthermore, several models for the mechanisms driving long COVID have been proposed; the biological drivers of long COVID seem to include both upstream pathobiological processes (perturbations of the immune system, coagulation system, mitochondrial activity) and downstream physiological changes (like end-organ tissue damage or neuro-/endotheliopathy) [4,5].

Lymphopenia and neutrophilia have been consistently associated with adverse outcomes in COVID-19, reflecting immune dysregulation and systemic inflammation, while elevated PLCR highlights thrombotic risk and increased CRP indicates systemic inflammatory burden.

This study aims to investigate the relationship between various immunological and inflammatory markers and the clinical course of elderly patients admitted with COVID-19. Analyzed markers include lymphocytes (LYM#), neutrophils (NEU#), C-reactive protein, platelet-large cell ratio (PLCR), and other parameters related to the inflammatory response. In addition, demographic factors such as gender and admission department are considered to assess the influence on discharge status and mortality. Thus, this research aims to provide a clearer framework for the early identification of high-risk patients using these immunological markers to optimize medical interventions and improve clinical outcomes. Special attention is given to investigating correlations between inflammatory markers and COVID-19 severity, with the aim of developing predictive models to help risk stratification. These results may have direct implications in the clinical management of elderly patients, providing essential data to guide early therapeutic and interventional decisions. Thus, this study aims not only to improve the prognosis of individual patients but also to optimize hospital resources by identifying patients at increased risk of poor outcomes.

## 2. Material and Methods

### 2.1. Study Design and Population

This study included 1429 elderly patients (aged >60 years) admitted with a confirmed diagnosis of COVID-19 between March 2020 and August 2022 at the “St. Apostol Andrei” County Emergency Clinical Hospital of Galati, Romania. Patients were monitored based on a complex set of clinical and laboratory data. The research was approved by the Hospital Ethics Committee (approval code: 29956, date: 25 November 2022) and was conducted in line with both local regulations and the Declaration of Helsinki.

### 2.2. Laboratory Data

Laboratory data included critical variables such as lymphocytes (LYM), neutrophils (NEU), platelet-large cell ratio (PLCR), and C-reactive protein (CRP). Reference ranges were based on the standards of the hospital laboratory, consistent with international guidelines. Not all patients had all tests available due to clinical constraints or diagnostic needs. Missing data were addressed using methods such as multiple imputation, ensuring robust interpretation of results. COVID-19 diagnosis was established based on RT-PCR (real-time reverse transcription-polymerase chain reaction) testing for SARS-CoV-2, complemented by chest CT imaging when necessary. Patients with incomplete medical records, those younger than 60 years, and those transferred from other hospitals without baseline laboratory data were excluded. Laboratory analyses were performed using a standard automated hematology analyzer for complete blood count parameters (LYM, NEU, and PLCR) and immunoturbidimetric assay for CRP measurement.

### 2.3. Variables

The discharge status of patients was classified into five categories:

Deceased—the patient did not survive the infection or COVID-19-related complications.
Aggravated—the condition worsened during hospitalization without improvement.
Stationary—no significant improvement or deterioration was observed until discharge.
Improved—partial recovery was observed with persisting symptoms.
Cured—the patient made a full recovery and was discharged without major complications.

For regression analysis, mortality was also treated as a binary variable (yes/no).

Independent variables and reference ranges were as follows:

- Lymphocytes (LYM#, 1–3.6 × 10^3^/µL);
- Neutrophils (NEU#, 2–6.3 × 10^3^/µL);
- Platelet-large cell ratio (PLCR, 13–43%);
- C-reactive protein (CRP, 0–10 mg/L);
- Gender;
- Requesting specialty (department of admission, e.g., ICU, internal medicine, or oncology).

### 2.4. Statistical Analysis

Normality of distribution was tested using the Kolmogorov–Smirnov test. Continuous variables were expressed as mean ± SD or median (IQR), while categorical variables were expressed as frequencies (%). Comparisons between groups were performed using Chi-square or Fisher’s exact test for categorical variables and the independent t-test or Mann–Whitney U test for continuous variables. Correlations were assessed using Pearson or Spearman coefficients. Logistic regression was applied to evaluate predictors of mortality. A *p*-value < 0.05 was considered statistically significant. Analyses were performed using IBM SPSS Statistics 20.

### 2.5. Ethics Approval

The study protocol was approved by the Ethics Committee of the “St. Apostol Andrei” County Emergency Clinical Hospital of Galati, Romania (approval code: 29956). All procedures were in accordance with the Declaration of Helsinki.

## 3. Results

Baseline characteristics of the study cohort are summarized in Table 1. The analysis included 1429 elderly patients admitted with COVID-19, with a mean age of 74.81 years. Male patients represented 53.4% of the population. At discharge, 54% of patients were classified as improved, 12.2% as stationary, 0.8% as aggravated, 1.7% as cured, and 31.3% as deceased. These characteristics provide a comprehensive overview of the population and serve as a basis for subsequent statistical analyses.

As shown in Table 2, significant differences were observed in the distribution of laboratory markers across discharge categories. Patients who died had the lowest lymphocyte counts, reflecting a profound impairment of the adaptive immune response, coupled with the highest neutrophil values, indicative of severe systemic inflammation. Elevated PLCR among deceased patients supports the hypothesis that platelet activation and thrombotic mechanisms are major contributors to COVID-19-related mortality. In contrast, patients who improved or were cured exhibited higher lymphocyte levels and lower inflammatory marker concentrations, underscoring the protective effect of preserved immune function. CRP followed a similar trend, with markedly increased values in patients with unfavorable outcomes, further highlighting the role of hyperinflammation in driving disease severity.

### 3.1. Significance of Discharge Status Variable

**Figure 1:**
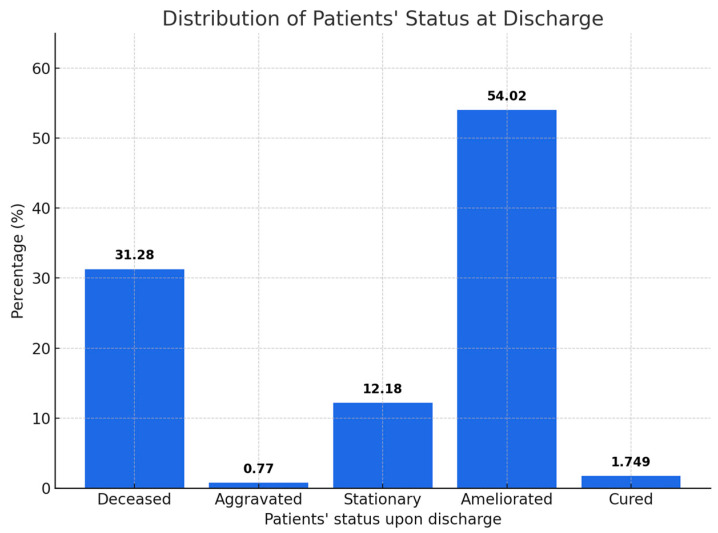
Distribution of discharge status categories. Flowchart of patient selection. From the total of 1540 patients initially admitted with a diagnosis of COVID-19, 111 were excluded due to incomplete laboratory data, leaving 1429 eligible patients included in the final analysis.

The graph in Figure 1 illustrates the distribution of discharge status across the study cohort. More than half of the patients experienced clinical improvement during hospitalization, while nearly one third of the cohort died, highlighting the severe burden of COVID-19 in the elderly. Only a minority of patients were discharged as fully cured, whereas a small proportion remained stationary or worsened during admission. These findings emphasize the heterogeneity of clinical evolution and underline the need to identify reliable prognostic markers. The highest proportion of patients, 54%, were discharged with a discharge status of “Improved”, indicating a significant improvement in health status. Approximately 31.3% of patients died, representing a significant proportion of cases. A smaller proportion, 12.2%, remained in a “Stationary” state, and only 1.7% of patients were completely cured. A very small number of patients, 0.8%, had a worsened status at discharge.

### 3.2. Complete Blood Cells Count Results

The analysis of the elderly patients with COVID-19 showed mean lymphocyte (LYM#) values slightly below the normal limit (1 × 10^3^–3.6 × 10^3^/µL), suggesting lymphopenia, a condition commonly seen in patients with severe viral infections. In contrast, the mean neutrophil count (NEU#), 8.441/µL, considerably exceeded the normal range (2 × 10^3^–6.3 × 10^3^/µL), indicating neutrophilia, an important marker of severity of infection. The platelet-large cell ratio (PLCR) was within normal limits (13–43%), while C-reactive protein averaged 92.094 mg/L, which is well above the normal range (0–10 mg/L), reflecting severe systemic inflammation (Table 3).

LYM# (1–3.6 × 10^3^/µL)—minimum and maximum values: 0.01/µL (min) and 56.92/µL (max). There is considerable variation in the values, which highlights patients with extremely low lymphocytes and others with very high levels, possibly indicating different stages of the immune response. Mean: 1.31/µL. The mean value is slightly lower than the median of the normal range (1 × 10^3^–3.6 × 10^3^/µL), which may indicate lymphopenia, commonly seen in COVID-19 patients. Standard deviation: 2.22. This value indicates high variability in lymphocyte values.

NEU# (2–6.3 × 10^3^/µL)—minimum and maximum values: 0.23 × 10^3^/µL (min) and 59.13 × 10^3^/µL (max). Neutrophils show a wide range of values, from extremely low to very high, suggesting either immunosuppression or a massive inflammatory response. Mean: 8.44 × 10^3^/µL. The mean is higher than the normal range (2 × 10^3^–6.3 × 10^3^/µL), indicating neutrophilia, a condition commonly seen in patients with severe infections. Standard deviation: 5.66. This value suggests moderate to high variability in neutrophil counts.

PLCR (13–43%)—minimum and maximum values: 7.60% (min) and 62.90% (max). This is a wide range of values, with some considerably exceeding the normal range, which may indicate large platelets and increased bone marrow activity. Mean: 28.12%. The mean value is within the normal range (13–43%). Standard deviation: 8.95. This value represents moderate variability.

C-reactive protein (CRP) (0–10 mg/L)—minimum and maximum values: 0.60 mg/L (min) and 450.50 mg/L (max). This is an extremely wide range of values, from almost normal to extremely elevated, suggesting severe inflammation in some patients. Mean: 92.09 mg/L. This value is well above the normal range (0–10 mg/L), indicating severe systemic inflammation in most patients. Standard deviation: 79.49. This value indicates extremely high variability of CRP values.

The test results in Table 4 indicate significant deviations from the normal distribution for all analyzed variables. The significant value, asymp. Sig. (2-tailed), is less than 0.05 for each variable, which suggests that the distributions are not normal according to the Kolmogorov–Smirnov test. This is also supported by the Kolmogorov–Smirnov Z values, which are high for each variable.

Although logistic regression does not require normally distributed predictors, certain laboratory variables (e.g., CRP) presented highly skewed distributions. For descriptive purposes and to facilitate visualization, these variables were log-transformed. However, logistic regression models were also run with untransformed values, yielding consistent results, as shown in Table 5.

On the basis of the Kolmogorov–Smirnov test, all four variables (LYM, NEU, P-LCR, and C-reactive protein) appear to follow a normal distribution. The *p*-values are very high (close to 1), suggesting that there is no significant deviation from normality. It can therefore be considered that these variables have a normal distribution and are suitable for the application of parametric statistical tests, if necessary.

### 3.3. Association Between Requesting Specialty and Discharge Status in Elderly Patients

The use of the Chi-square test to assess the association between discharge status and requesting specialty is justified because both variables involved are categorical, which makes this test suitable for analyzing relationships between discrete variables. The Chi-square test allows us to test the hypothesis of association between the distribution of discharge status and inpatient departments to assess whether there are significant differences between them. In addition, the results may help to identify the potential influences of admission departments on the clinical course of elderly patients with COVID-19, providing important insights into how different medical departments affect treatment outcomes (Table 6).

Table 6 highlights the distribution of elderly patients with COVID-19 according to the department in which they were admitted and their death status. In the internal medicine department, out of 793 patients, 507 survived and 286 died, representing the department with the highest number of deaths. In the medical oncology department, out of 92 patients, 31 died, indicating a high mortality rate (about 33.7%).

The departments of thoracic surgery and diabetes, nutrition, and metabolic diseases also had a significant number of deaths, with 16 out of 72 and 23 out of 79 patients dying, respectively. In contrast, endocrinology and rheumatology had a low number of deaths, namely 0 out of 3 patients in endocrinology and 1 out of 31 in rheumatology.

Thus, the numerical distribution shows that departments with more critically ill patients, such as internal medicine and medical oncology, had the highest mortality rates, suggesting an association between department type and death risk.

The Chi-square test (Chi-square) indicates a significant association between the requesting specialty and death status in elderly COVID-19 patients, with a Pearson Chi-square test value of 41.448, with 9 degrees of freedom and a significance of 0.000, suggesting a statistically significant relationship (*p* < 0.05). This means that there are important differences in the distribution of deaths according to the department where the patients were admitted (Table 7).

The Chi-square test assumes independence of observations and an expected frequency of at least 5 in each cell. All assumptions were checked and fulfilled before applying the test.

The value of the Likelihood Ratio test is also significant, which reinforces the conclusion that there is a relationship between department and probability of death. The linear association is also significant (*p* < 0.001), indicating a possible linear trend between the variables.

The symmetric measures show the strength of the relationship between the variables “Requesting specialty” and “Deceased”. The Phi value is 0.170, and Cramer’s V is also 0.170, both indicating a weak relationship between the two variables (based on the Cramer’s V scale, where 0.1–0.3 indicates a weak relationship).

The approximate significance (Sig.) is 0.000, which means that this relationship is statistically significant (*p* < 0.05). In other words, even if the strength of the association is weak, the association between the requesting specialty and the probability of death is statistically significant (Table 8).

### 3.4. Analyzing Correlations Between Study Variables: The First Step in Demonstrating Causal Relationships

The main aim of this study is to identify the relationships between immunological and inflammatory markers (LYM, NEU, P-CRP, and C-reactive protein) and dependent variables, such as discharge status and deceased, to better understand how these factors may influence the clinical course in elderly patients with COVID-19.

To analyze these relationships, we decided to use the Spearman correlation test. The choice to use this test was justified by the characteristics of the variables involved. Although immunological and inflammatory markers are normally distributed, the dependent variables, such as discharge status and deceased, are ordinal and nominal, respectively. Spearman’s correlation is appropriate in such cases because it does not require strict assumptions about the distribution of variables and can assess relationships between numeric and ordinal variables. This method provides a robust assessment of the associations between biological markers and patients’ clinical course, allowing for the identification of significant correlations that can contribute to risk modeling and optimization of clinical decisions.

Table 9 presents the Spearman correlations between the variables “Deceased”, “Discharge Status”, and various biological markers, such as LYM, NEU, PLCR, C-reactive protein, gender and requesting specialty. Statistically significant correlations are marked with two levels of significance: *p* < 0.01 and *p* < 0.05.

The variables “Deceased” and “LYM” have a weak negative correlation (−0.252), indicating that deceased patients tend to have lower lymphocyte levels. On the other hand, the correlation between “Deceased” and “NEU” is positive (+0.252), indicating an association between increased neutrophil counts and risk of death.

“Discharge status” has a weak positive correlation with “LYM” (+0.248), suggesting that patients with a favorable outcome tend to have a higher lymphocyte count. In contrast, the correlation between “Discharge status” and “NEU” is negative (−0.198), suggesting that an increased neutrophil count is associated with less favorable outcomes.

C-reactive protein shows significant positive correlations with “NEU” (+0.313) and “Deceased” (+0.299), indicating that increased levels of this inflammatory protein are associated with greater severity of disease and risk of death.

Overall, this table indicates that there are significant relationships between various immunological markers and the course of elderly patients with COVID-19, suggesting that these markers may play an important role in predicting clinical outcomes.

### 3.5. Assessment of Significant Differences Between Groups Based on Dependent Variables

Purpose: To compare the values of immunological continuous variables (LYM, NEU, PLCR, and C-reactive protein) between different categories of ordinal dependent variables such as “Deceased” and “Discharge Status”. Through this comparison, we aim to identify potential predictive biomarkers of disease severity that may help to optimize treatment strategies.

The methodological applicability of the Kruskal–Wallis test is justified by the non-parametric nature of the analyzed data. Since the values of these markers do not abide by the assumption of normality, Kruskal–Wallis is an ideal test for assessing significant differences between groups for continuous variables. It analyses between-group differences without relying on the normal distribution of the data and is useful in comparisons between multiple groups, such as different stages of discharge or death status. This method has high practical value in clinical trials as it can detect critical variations in inflammatory and immunological biomarkers, providing clues for patient prognosis and helping to improve therapeutic approaches (Table 10).

The results of the Kruskal–Wallis test show that there are significant differences between the values of immunological and inflammatory markers (LYM, NEU, PLCR, and C-reactive protein) according to the patients’ evolution, as measured by discharge status. The asymptotic significance values (*p*-values) for all markers are below the 0.05 threshold, indicating that variations in these markers are significantly associated with patient outcome (deceased, worsened, stationary, improved, or cured). Thus, these markers can be considered important predictors of clinical evolution in elderly patients with COVID-19 (Table 11).

The results of the Kruskal–Wallis test indicate that the values of immunological and inflammatory markers (LYM, NEU, PLCR, and C-reactive protein) show significant differences between patients who died and those who survived. Asymptotic significance values (*p*-values) are all below 0.05, confirming that these markers are significantly associated with death in elderly patients with COVID-19. These findings suggest that these markers can be used as predictors of the risk of death in elderly patients affected by COVID-19.

### 3.6. Analyzing the Prediction of Discharge Status Using Logistic Regression

Logistic regression is essential to demonstrate the link between immunological and inflammatory markers and the risk of death or clinical course of elderly patients with COVID-19. It is a rigorous method to assess and quantify the likelihood that these markers are predictors of outcome, thus allowing the research hypothesis stated in the title of this paper to be supported.

The most important step in identifying the risk of death in elderly patients with COVID-19, using logistic regression, is to determine the independent variables that have the greatest influence on death. In the context of the study based on immunological and inflammatory markers, variables such as C-reactive protein, LYM, NEU, and PLCR play a critical role in estimating the risk of death.

Logistic regression is ideal for situations in which the dependent variable is binomial or dichotomous (as in the case of the deceased variable with two categories: yes and no). This method allows for the assessment of the relationship between one or more independent variables and the probability of a particular event (in this case, death) to happen.

The “Case Processing Summary” data in Table 12 indicates that the analysis included only 634 cases, representing 44.4% of the total of 1429, while the remaining 55.6% of cases were not included due to missing data. This significant proportion of missing cases may influence the results and the validity of the conclusions. However, there were no cases that were intentionally not included, so all available data, apart from incomplete data, were taken into account. This suggests the need to consider additional methods for handling missing data in the analysis.

Table 13 shows the performance of the logistic regression model at stage 0, i.e., before the predictor variables are added to the model. At this stage, the model only includes the constant and makes a prediction based on the majority prevalence.

The model correctly predicts 100% of cases in which patients did not die, but it cannot correctly predict any deaths. This is reflected in the fact that all death cases are incorrectly classified. Overall, the model manages to achieve an overall accuracy of 68.8%, which indicates that the underlying decision is based solely on the fact that most patients did not die.

This step shows the need to add predictor variables to improve the model’s ability to distinguish between cases of deceased and surviving patients.

Table 14 shows the results for the initial logistic regression model, which contains only the constant with no predictor variables. The B-coefficient for the constant is negative (-0.789), suggesting that the probability of death (the dependent variable) is lower than the probability of survival in the absence of other predictors.

The statistical significance of the constant is very high (*p* < 0.001), indicating that the result is significant. Exp(B), also known as the “odds ratio”, is 0.454, which means that, with no other variables in the model, a patient’s chances of dying are lower than their chances of surviving. In other words, the original model suggests that patients are more likely to survive than to die, but without taking into account biological markers or other clinical factors.

Table 15 shows the variables that were not included in the original model, but which may have a significant influence on the prediction of the death risk. The score values indicate the degree to which each independent variable (ILYM, NEU, IN, PLCR, and C-reactive protein) could improve the model if included.

All variables have very high statistical significance (*p* < 0.001), indicating that each has the potential to contribute significantly to the logistic regression model. The overall statistics show that together, these variables make a significant contribution, suggesting that their inclusion would improve the ability of the model to predict the risk of death. This indicates that immunological and inflammatory markers could be valuable predictors in assessing the risk of death in elderly patients with COVID-19.

Table 16 shows the overall significance of the logistic regression model. The Chi-square indicates whether the addition of explanatory variables significantly improves the ability of the model to predict the dependent variable which, in this case, is the risk of death in elderly patients with COVID-19.

The results show a significant Chi-square value (*p* < 0.001), which indicates that the model with included variables better explains the variability in the data than a model without these variables. This suggests that immunological and inflammatory variables have a significant influence on the risk of death and improve model prediction.

Table 17 evaluates the performance of the logistic regression model in correctly predicting deaths. The model makes predictions with 92.2% accuracy for patients who did not die, indicating a good ability to identify survivors. However, the model’s accuracy in correctly predicting deaths is much lower, at only 26.3%. Overall, the model correctly predicts 71.6% of cases.

This suggests that although the model is more effective at predicting survival, it has difficulty correctly identifying patients who have died, which may indicate the need for improvements in predictor variables or model adjustment.

The logistic regression analysis in Table 18 shows the influence of each variable on the risk of death in elderly patients with COVID-19 based on immunological and inflammatory variables.

LYM (lymphocytes) have a negative coefficient (−0.156), indicating that an increase in lymphocyte count reduces the risk of death. The value Exp(B) = 0.856 shows that each unit increase in LYM decreases the risk of death by about 14.4%. This result is highly statistically significant (*p* < 0.001).

NEU (neutrophils) have a positive coefficient (0.070), which means that an increase in neutrophil counts increases the risk of death. The value Exp(B) = 1.072 indicates that each increase in neutrophils is associated with an increase in the risk of death by 7.2%. This is also a highly significant relationship (*p* < 0.001).

The PLCR (platelet-large cell ratio) has a positive coefficient (0.024), suggesting that an increase in this parameter increases the risk of death. The value Exp(B) = 1.024 shows a small increase of 2.4% in the risk of death for each unit increase in PLCR, and this association is significant (*p* < 0.026).

C-reactive protein has a very small (0.004) but statistically significant (*p* < 0.002) positive coefficient, suggesting that increases in C-reactive protein are associated with a marginal increase in the risk of death. The value Exp(B) = 1.004 indicates a 0.4% increase in the risk of death for each unit increase in C-reactive protein.

The constant data has a negative coefficient (−2.363), which indicates the probability of death in the absence of any influence of the independent variables, with Exp(B) = 0.094, suggesting a very low probability of death under these conditions.

In conclusion, the variables LYM, NEU, PLCR, and C-reactive protein have a significant influence on the risk of death, with lymphocytes having a protective effect and neutrophils, PLCR, and C-reactive protein being associated with an increased death risk.

## 4. Discussion

Our findings confirm the trends reported in post-2021 literature, which consistently highlight inflammatory and coagulation markers as central predictors of disease severity and mortality in elderly COVID-19 patients, reinforcing their role in clinical risk stratification and therapeutic decision making.

Lymphocytes (LYM#), neutrophils (NEU#), C-reactive protein, and PLCR were analyzed to determine whether they are significant predictors of disease severity and mortality. Each of the inflammatory markers monitoring in COVID-19 may provide evidence for specificity in predicting the severity, mortality, and need for intensive care treatments among patients infected with SARS-CoV-2 [6].

The mean LYM# of 1.310/µL, below normal, indicates lymphopenia, a phenomenon commonly associated with COVID-19 infection. Previous studies show that lymphopenia is a common marker in patients with severe forms of the disease. Wang and colleagues [7] reported lymphopenia in the majority of patients hospitalized with COVID-19, associating this condition with an unfavorable prognosis. These data are also confirmed in our study, in which low lymphocytes are negatively correlated with the risk of death, suggesting a protective role.

In contrast, neutrophilia was predominant among patients, with the mean NEU# being 8.441/µL, well above the normal range. Neutrophilia is a well-known marker of severe inflammation and infection. Zhang and co-workers [8] demonstrated that an increased neutrophil count is associated with accelerated disease progression and increased risk of complications. Similarly, our results show a positive correlation between neutrophils and risk of death, confirming their role as a risk factor for COVID-19 mortality.

C-reactive protein (CRP), another inflammatory marker, showed extremely high values in this study (mean: 92.094 mg/L), suggesting severe systemic inflammation in most patients. These values are consistent with the literature, which recognizes CRP as an important predictor of disease severity. Chen et al. [9] reported that elevated CRP levels are directly correlated with mortality in patients with COVID-19. Our results also show a significant correlation between CRP and death, emphasizing the importance of this marker in assessing the risk of fatal complications.

Other research results focused on the relationship of these biological parameters with the hospitalization duration, need for ICU admittance, or predictors of neuro-psychiatric manifestations, with some showing that during the onset of COVID-19 disease, a polarization of lymphocytes to departments and a memory phenotype occur [10,11,12,13].

In terms of inpatient departments, patients in internal medicine and oncology had the highest mortality rates. This reflects the serious condition and complexity of cases treated in these departments. Richardson et al. [14] emphasized the vulnerability of patients with chronic comorbidities, such as cardiovascular disease or cancer, to COVID-19. The high mortality in these departments indicates that pre-existing conditions play a major role in increasing the risk of death.

Correlation analyses showed significant relationships between inflammatory markers and discharge status or death. For example, an increase in lymphocyte count was associated with a favorable outcome, while an increase in neutrophils and C-reactive protein was correlated with an increased risk of death. These findings are supported by Gómez et al. [15], who demonstrated that inflammatory markers are strong predictors of COVID-19 severity. A clinical assessment of COVID-19 cases remains key to decision making, but laboratory biomarkers enable important information to be obtained, assisting in decision making and providing data regarding underlying biological processes [15,16,17,18].

Logistic regression confirmed that LYM#, NEU#, PLCR, and C-reactive protein had a significant influence on the risk of death. Lymphocytes had a protective effect, reducing the risk of death by 14.4%, while neutrophils and C-reactive protein increased the risk of death. These results emphasize the importance of evaluating these markers in predicting the clinical course of elderly patients with COVID-19. Extended research proved that COVID-19 may lead to hyperinflammatory response and “cytokine storm” and tissue damage via apoptosis and pyroptosis. Higher C-reactive protein (CRP) levels are associated with disease progression and mortality [18,19].

In our study, variations in immunological and inflammatory markers indicated a wide spectrum of severity of clinical conditions specific to COVID-19 patients. The internal medicine and neurology departments had the most patients, and this emphasizes the prevalence of chronic and complex conditions in this sample. Discharge status indicated that 54% of patients were discharged with an improving status, while 31.3% died, indicating a high mortality rate. Despite the fact that multiple biomarkers of unfavorable outcomes in COVID-19 have been studied, many of them are expensive or even unavailable in most types of clinical practice. Also, the predictive abilities of some biomarkers are not high enough. These factors make some laboratory parameters secondary for predicting unfavorable outcomes. Therefore, our focus was specific to lab tests that are usually available and their correlations with disease evolution.

Spearman correlations revealed significant relationships between immunological markers and patients’ evolution. The negative correlation between lymphocytes and death (−0.252) suggests that patients with low lymphocytes have a higher risk of death, while the positive correlation between neutrophils and death (+0.252) indicates that patients with elevated neutrophils have a higher risk of mortality. C-reactive protein also showed a positive correlation with the risk of death (+0.299), confirming its role as a marker of severe inflammation.

The results of the Kruskal–Wallis test showed significant differences between the values of immunological and inflammatory markers (LYM, NEU, PLCR, and C-reactive protein) according to the patients’ evolution as measured by discharge status. Asymptotic significance values (*p*-value) less than 0.05 indicate that variations in these markers are closely related to patient outcome, whether improvement, stagnation, or death.

Biomarker analysis of inflammatory biomarkers like CRP, LDH, and the cytokines IL-6, IL-10, and TNFα, alongside a thorough clinical assessment of COVID-19 patients, enables more accurate stratification of high and low-risk cases and the need for intensive care support [19,20,21,22,23].

Logistic regression, performed to assess the death risk, confirmed that immunological and inflammatory markers are significant predictors of mortality. Negative coefficients for lymphocytes (LYM) (−0.156) indicate that an increase in lymphocytes reduces the death risk, while positive coefficients for neutrophils (NEU) (+0.070), PLCR (+0.024), and C-reactive protein (+0.004) suggest that increased values of these markers are associated with a higher risk of death. These results demonstrate a significant relationship between these markers and the likelihood of death in elderly patients with COVID-19. Interestingly, investigation into the temporal dynamics of identified auto-antibodies revealed a proportion of antibodies that were likely present pre-infection (including those against IFNα2 and IL1β), illustrating the complex nature of whether pre-existing autoantibodies increase susceptibility to infection and progression of COVID-19 or whether they result from the infection itself [2,18,23,24].

Thus, our study highlights that immunological and inflammatory markers are potential predictors of the risk of death in elderly patients with COVID-19, contributing to a better understanding of how these patients respond to the disease and the adjustment of therapeutic strategies.

### 4.1. Limitations of the Study

This study has several limitations that should be acknowledged. First, all biomarkers were collected at a single timepoint (admission), which may not fully capture the dynamic changes in inflammatory and immune responses throughout hospitalization. Second, we did not perform stratified analyses according to age groups within the elderly population or adjust for the presence of specific comorbidities, such as diabetes or cardiovascular disease, which are known to influence COVID-19 outcomes. These factors may have acted as residual confounders, potentially affecting the strength of the observed associations. Third, our findings should be interpreted in the context of single-center data, which might limit generalizability. Nevertheless, the results are consistent with more recent evidence published after 2021, which also highlights the prognostic role of hematological and inflammatory biomarkers in COVID-19 patients. Future multicenter and longitudinal studies are needed to validate these findings and assess their clinical utility in risk stratification.

### 4.2. PLCR and Thrombotic Mechanisms

One of the most interesting findings of our study is the association between elevated platelet-large cell ratio (PLCR) and mortality in elderly COVID-19 patients. PLCR reflects platelet size heterogeneity and the presence of larger, more reactive platelets, which are known to have greater pro-thrombotic potential. In the context of COVID-19, endothelial dysfunction, systemic inflammation, and cytokine release contribute to platelet activation and aggregation, thus favoring microvascular thrombosis. This pathophysiological cascade may explain why patients with higher PLCR values have an increased risk of unfavorable outcomes. Similar observations have been reported in other inflammatory and infectious diseases, as well as in cardiovascular pathology, supporting the hypothesis that PLCR could serve as a useful surrogate marker for thrombotic risk. Expanding research in this direction could improve our ability to identify patients at higher risk and guide anticoagulant strategies.

## 5. Conclusions

The study emphasized a significant association between immunological and inflammatory markers and the clinical course of elderly patients with COVID-19. The results showed that low lymphocyte counts (LYM) are associated with a higher risk of death, suggesting that lymphopenia is an important predictor of disease severity. Also, increased neutrophil counts (NEU) were associated with a negative evolution, indicating a central inflammatory role in severe cases of COVID-19.

C-reactive protein, known to reflect systemic inflammation, had greatly elevated values in patients who died, emphasizing the importance of inflammation in the fatal course of the disease. PLCR was also associated with an increased risk of death, suggesting that thrombosis plays an important role in the severe course of COVID-19.

Immunological and inflammatory markers play a key role in predicting clinical evolution in elderly patients with COVID-19. Analysis and constant monitoring of these parameters are crucial for the early identification of patients at increased risk of severe complications and death. Thus, these markers not only provide a detailed picture of the severity of health status but also help to guide clinical decisions. In this context, the use of these data can underpin the development of personalized therapeutic strategies to optimize treatments and significantly improve the chances of survival of critically ill patients.

## Figures and Tables

**Table 1:** Baseline characteristics of the study population (n = 1429).

Characteristic	Value (n, %) or Mean ± SD
Age, years (mean ± SD)	74.81, 7.704
Sex, male (%)	764, 53.4%
Discharge status: improved (%)	772, 54%
Discharge status: stationary (%)	174, 12.2%
Discharge status: aggravated (%)	11, 0.8%
Discharge status: deceased (%)	447, 31.3%
Discharge status: cured (%)	25, 1.7%

Note: Data are presented as mean ± standard deviation (SD) for continuous variables and as number (percentage) for categorical variables.

**Table 2:** Comparison of laboratory markers by discharge status.

Variable	Deceased (n = 447)	Aggravated (n = 11)	Stationary (n = 174)	Improved (n = 772)	Cured (n = 9)	Kolmogorov–Smirnov *p*-Value
Lymphocytes (LYM, ×10^3^/µL)	0.546, 2.33	-	1.009, 2.312	1.455, 2.116	2.013, 1.398	0.200 *
Neutrophils (NEU, ×10^3^/µL)	10.566, 5.721	-	8.180, 4.480	8.054, 5.282	8.684, 3.784	0.200 *
Platelet-large cell ratio (PLCR, %)	30.683, 8.693	-	27.008, 8.323	28.294, 8.532	24.559, 8.789	0.200 *
C-reactive protein (CRP, mg/L)	112.791, 75.037	-	84.625, 85.726	77.018, 76.928	18.660, 86.666	0.200 *

Notes: Values are expressed as mean ± SD for normally distributed variables and as median (IQR) for skewed distributions. *p*-values were obtained by ANOVA or Kruskal–Wallis test, as appropriate. Abbreviations: LYM—lymphocytes; NEU—neutrophils; PLCR—platelet-large cell ratio; CRP—C-reactive protein. * This is a lower bound of the true significance.

**Table 3:** Descriptive statistics for numeric variables.

	N	Min	Max	Mean		Std Dev	Variance
Descriptive Statistics	Statistic	Statistic	Statistic	Statistic	Std. Error	Statistic	Statistic
LYM# 1 × 10^3^–3.6 × 10^3^/µL	1127	0.01	56.92	1.31	0.07	2.22	4.93
NEU# 2 × 10^3^–6.3 × 10^3^/µL	1128	0.23	59.13	8.44	0.17	5.66	32.04
PLCR 13–43% PLCR 13–43	1166	7.60	62.90	28.12	0.26	8.95	80.16
C-Reactive Protein 0–10 mg/L	780	0.60	450.50	92.09	2.85	79.49	6318.27
Valid N (listwise)	67						

**Table 4:** Distribution tests for numeric variables.

One-Sample Kolmogorov–Smirnov Test		LYM# 1–3.6 × 10^3^-/µL	NEU# 2–6.3 × 10^3^-/µL	PLCR 13–43%	C-Reactive Protein 0–10 mg/L
N		1127	1128	1166	780
Normal Parameters ^a,b^	Mean	1.310	8.441	28.121	92.094
	Std. Dev.	2.221	5.660	8.953	79.488
Most Extreme Differences	Absolute	0.305	0.127	0.048	0.125
	Positive	0.254	0.127	0.048	0.100
	Negative	−0.305	−0.111	−0.027	−0.125
Kolmogorov–Smirnov Z		10.224	4.278	1.653	3.487
Asymp. Sig. (2-tailed)		0.000	0.000	0.008	0.000

a. Test distribution is normal. b. Calculated from data.

**Table 5:** Transformed variable distribution tests.

One-Sample Kolmogorov–Smirnov Test		LYM	NEU	P-LCR	C-Reactive Protein
N		1126	1127	1165	779
Normal Parameters ^a,b^	Mean	1.310	8.453	28.124	92.280
	Std. Dev.	2.207	5.622	8.911	79.119
Most Extreme Differences	Absolute	0.007	0.005	0.005	0.013
	Positive	0.007	0.005	0.005	0.013
	Negative	−0.007	−0.003	−0.005	−0.012
Kolmogorov–Smirnov Z		0.233	0.156	0.186	0.368
Asymp. Sig. (2-tailed)		1.000	1.000	1.000	0.999

a. Test distribution is normal. b. Calculated from data.

**Table 6:** Contingency table between the variables “Deceased” and “Requesting specialty”.

Requesting Specialty × Deceased Crosstabulation		Deceased		Total
		No	From	
Requesting specialty	Cardiology	75	11	86
	Thoracic Surgery	56	16	72
	Endocrinology	3	0	3
	Geriatrics and Gerontology	15	5	20
	Nephrology	32	20	52
	Rheumatology	30	1	31
	Diabetes, Nutrition, and Metabolic Diseases	56	23	79
	Internal Medicine	507	286	793
	Neurology	147	54	201
	Medical Oncology	61	31	92
Total		982	447	1429

**Table 7:** Chi-square tests between the variables “Deceased” and “Requesting specialty”.

Chi-Square Tests	Value	df	Asymp. Sig. (2-Sided)
Pearson Chi-Square	41.448 ^a^	9	<0.001
Likelihood Ratio	49.359	9	<0.001
Linear-by-Linear Association	16.202	1	<0.001
N of Valid Cases	1429		

a. Two cells (10.0%) have expected count less than 5. The minimum expected count is 0.94.

**Table 8:** Association relationship between the variables “Deceased” and “Requesting specialty”.

Symmetric Measures		Value	Approx. Mr Sig.
Nominal by Nominal	Phi	0.170	0.000
	Cramer’s V	0.170	0.000
N of Valid Cases		1429	

**Table 9:** Spearman correlations.

Correlations			Deceased	Discharge Status	LYM	NEU	PLCR	C-Reactive Protein
Spearman’s rho	Deceased	C.C.	1.000	−0.892 **	−0.252 **	0.252 **	0.136 **	0.299 **
		Sig. (2-tailed)		0.000	0.000	0.000	0.000	0.000
		N	1429	1429	1126	1127	1165	779
	Discharge Status	C.C.	−0.892 **	1.000	0.248 **	−0.198 **	−0.086 **	−0.303 **
		Sig. (2-tailed)	0.000		0.000	0.000	0.003	0.000
		N	1429	1429	1126	1127	1165	779
	LYM	C.C.	−0.252 **	0.248 **	1.000	−0.097 **	−0.138 **	−0.218 **
		Sig. (2-tailed)	0.000	0.000		0.001	0.000	0.000
		N	1126	1126	1126	1125	1125	635
	NEU	C.C.	0.252 **	−0.198 **	−0.097 **	1.000	0.126 **	0.313 **
		Sig. (2-tailed)	0.000	0.000	0.001		0.000	0.000
		N	1127	1127	1125	1127	1126	634
	PLCR	C.C.	0.136 **	−0.086 **	−0.138 **	0.126 **	1.000	0.062
		Sig. (2-tailed)	0.000	0.003	0.000	0.000		0.118
		N	1165	1165	1125	1126	1165	640
	C-Reactive Protein	C.C.	0.299 **	−0.303 **	−0.218 **	0.313 **	0.062	1.000
		Sig. (2-tailed)	0.000	0.000	0.000	0.000	0.118	
		N	779	779	635	634	640	779
	Gender	C.C.	−0.015	0.008	0.049	−0.077 **	0.004	−0.073 *
		Sig. (2-tailed)	0.566	0.772	0.098	0.009	0.904	0.041
		N	1429	1429	1126	1127	1165	779
	Requesting Section	C.C.	0.066 *	−0.119 **	−0.037	0.021	0.014	−0.003
		Sig. (2-tailed)	0.012	0.000	0.216	0.474	0.643	0.935
		N	1429	1429	1126	1127	1165	779

** Correlation is significant at the 0.01 level (2-tailed). * Correlation is significant at the 0.05 level (2-tailed). C.C. = correlation coefficient.

**Table 10:** Kruskal–Wallis test results for the independent variables and the discharge status variable.

Test Statistics ^a,b^	LYM	NEU	PLCR	C-Reactive Protein
Chi-Square	76.382	77.087	36.674	75.069
df	4	4	4	4
Asymp. Sig.	0.000	0.000	0.000	0.000

a. Kruskal–Wallis test. b. Grouping Variable: discharge status.

**Table 11:** Kruskal–Wallis test results for the independent variables and the deceased variable.

Test Statistics ^a,b^	LYM	NEU	PLCR	C-Reactive Protein
Chi-Square	71.639	71.266	21.406	69.714
df	1	1	1	1
Asymp. Sig.	0.000	0.000	0.000	0.000

a. Kruskal–Wallis test. b. Grouping Variable: deceased.

**Table 12:** Number of patients included in the logistic regression equation.

Case Processing Summary			
Unweighted Cases ^a^		N	Percentage
Selected Cases	Included in Analysis	634	44.4
	Missing Cases	795	55.6
	Total	1429	100.0
Unselected Cases		0	0.0
Total		1429	100.0

a. If weight is in effect, see classification table for the total number of cases.

**Table 13:** Model performance without predictor variables.

Classification Table ^a,b^ Block 0					
Observed			Predicted		
			Deceased		Percentage Correct
			No	From	
Step 0	Deceased	No	436	0	100.0
		From	198	0	0.0
	Overall Percentage				68.8

a. Constant is included in the model. b. The cut value is 0.500.

**Table 14:** Significance of the constant without predictors.

Variables in the Equation—Block 0							
		B	S.E.	Wald	df	Mr	Exp(B)
Step 0	Constant	−0.789	0.086	84.846	1	0.000	0.454

**Table 15:** Significance of predictor variables.

Variables Not in the Equation					
			Score	df	Mr
Step 0	Variables	INF_LYM	21.638	1	0.000
		INF_NEU	28.161	1	0.000
		INF_PLCR	11.864	1	0.001
		INF_ProteinC	28.853	1	0.000
	Overall Statistics		64.081	4	0.000

**Table 16:** Omnibus test—logistic regression.

Omnibus Tests of Model Coefficients				
		Chi-Square	df	Mr
Step 1	Step	66.931	4	0.000
	Block	66.931	4	0.000
	Model	66.931	4	0.000

**Table 17:** Logistic regression model accuracy and performance.

Classification Table ^a^					
Observed			Predicted		
			Deceased		Percentage Correct
			No	From	
Step 1	Deceased	No	402	34	92.2
		From	146	52	26.3
	Overall Percentage				71.6

a. The cut value is 0.500.

**Table 18:** Predictor variables of the regression model.

Variables in the Equation							
		B	S.E.	Wald	df	Mr	Exp(B)
Step 1 ^a^	LYM	−0.156	0.043	13.113	1	0.000	0.856
	NEU	0.070	0.018	15.878	1	0.000	1.072
	PLCR	0.024	0.011	4.972	1	0.026	1.024
	C-Reactive Protein	0.004	0.001	9.766	1	0.002	1.004
	Constant	−2.363	0.372	40.303	1	0.000	0.094

a. Variable(s) entered in step 1: LYM, NEU, PLCR, and C-reactive protein.

## Data Availability

No new data were created.

## References

[1] Huang C., Wang Y., Li X., Ren L., Zhao J., Hu Y., Zhang L., Fan G., Xu J., Gu X. (2020). Clinical features of patients infected with 2019 novel coronavirus in Wuhan, China. Lancet.

[2] Sweet D.R., Freeman M.L., Zidar D.A. (2023). Immunohematologic Biomarkers in COVID-19: Insights into Pathogenesis, Prognosis, and Prevention. Pathog. Immun..

[3] Grau M., Ibershoff L., Zacher J., Bros J., Tomschi F., Diebold K.F., Predel H.G., Bloch W. (2022). Even patients with mild COVID-19 symptoms after SARS-CoV-2 infection show prolonged altered red blood cell morphology and rheological parameters. J. Cell Mol. Med..

[4] Peluso M.J., Deeks S.G. (2024). Mechanisms of long COVID and the path toward therapeutics. Cell.

[5] Zhou F., Yu T., Du R., Fan G., Liu Y., Liu Z., Xiang J., Wang Y., Song B., Gu X. (2020). Clinical course and risk factors for mortality of adult inpatients with COVID-19 in Wuhan, China: A retrospective cohort study. Lancet.

[6] Weiss P., Murdoch D.R. (2020). Clinical course and mortality risk of severe COVID-19. Lancet.

[7] Wang D., Hu B., Hu C., Zhu F., Liu X., Zhang J., Wang B., Xiang H., Cheng Z., Xiong Y. (2020). Clinical Characteristics of 138 Hospitalized Patients with 2019 Novel Coronavirus-Infected Pneumonia in Wuhan, China. JAMA.

[8] Zhang C., Shi L., Wang F.S. (2020). Liver injury in COVID-19: Management and challenges. Lancet Gastroenterol. Hepatol..

[9] Chen N., Zhou M., Dong X., Qu J., Gong F., Han Y., Qiu Y., Wang J., Liu Y., Wei Y. (2020). Epidemiological and clinical characteristics of 99 cases of 2019 novel coronavirus pneumonia in Wuhan, China: A descriptive study. Lancet.

[10] Lescai A.-M., Anghele M., Baltă A.A., Dumitrache Anghele A., Dragomir L., Ciubara B. (2024). Addiction Patient’s Relationship to Self and Predictions on the Estimated Hospitalization Duration. BRAIN Broad Res. Artif. Intell. Neurosci..

[11] Lescai A.-M., Anghele M., Voineag C., Baltă A.A., Ciubara A. (2023). Mental Health and Environmental Coping Mechanisms of the Diabetic Patient and Implications on Hospitalization Duration. BRAIN Broad Res. Artif. Intell. Neurosci..

[12] Popazu C., Romila A., Petrea M., Grosu R.M., Lescai A.M., Vlad A.L., Oprea V.D., Baltă A.A.Ș. (2025). Overview of Inflammatory and Coagulation Markers in Elderly Patients with COVID-19: Retrospective Analysis of Laboratory Results. Life.

[13] Anghele M., Marina V., Moscu C.A., Romila A., Dragomir L., Anghele A.-D., Lescai A.-M. (2023). Use of the WBSI Questionnaire in a Study Group of Patients with Polytrauma During the Period 2015–2021. BRAIN Broad Res. Artif. Intell. Neurosci..

[14] Richardson S., Hirsch J.S., Narasimhan M., Crawford J.M., McGinn T., Davidson K.W., Barnaby D.P., Becker L.B., Chelico J.D., The Northwell COVID-19 Research Consortium (2020). Presenting Characteristics, Comorbidities, and Outcomes Among 5700 Patients Hospitalized with COVID-19 in the New York City Area. JAMA.

[15] Buonacera A., Stancanelli B., Colaci M., Malatino L. (2022). Neutrophil to Lymphocyte Ratio: An Emerging Marker of the Relationships between the Immune System and Diseases. Int. J. Mol. Sci..

[16] Keddie S., Ziff O., Chou M.K.L., Taylor R.L., Heslegrave A., Garr E., Lakdawala N., Church A., Ludwig D., Manson J. (2020). Laboratory biomarkers associated with COVID-19 severity and management. Clin. Immunol..

[17] Fernández-de-Las-Peñas C., Ryan-Murua P., de-la-Llave-Rincón A.I., Gómez-Mayordomo V., Arendt-Nielsen L., Torres-Macho J. (2022). Serological biomarkers of COVID-19 severity at hospital admission are not related to long-term post-COVID pain symptoms in hospitalized COVID-19 survivors. Pain.

[18] Skakun O., Vandzhura Y., Vandzhura I., Symchych K., Symchych A. (2024). Biomarkers for unfavourable outcomes prediction in COVID-19 patients: A narrative review. J. Emerg. Crit. Care Med..

[19] Luo X., Zhou W., Yan X., Guo T., Wang B., Xia H., Ye L., Xiong J., Jiang Z., Liu Y. (2020). Prognostic Value of C-Reactive Protein in Patients with Coronavirus 2019. Clin. Infect. Dis..

[20] Gambichler T., Schuleit N., Susok L., Becker J.C., Scheel C.H., Torres-Reyes C., Overheu O., Reinacher-Schick A., Schmidt W. (2023). Prognostic Performance of Inflammatory Biomarkers Based on Complete Blood Counts in COVID-19 Patients. Viruses.

[21] Dwivedi T., Raj A., Das N., Gupta R., Gupta N., Tiwari P., Sahoo B., Sagiraju H.K.R., Sirohiya P., Ratre B. (2023). The Evaluation of Laboratory Parameters as Predictors of Disease Severity and Mortality in COVID-19 Patients: A Retrospective Study from a Tertiary Care Hospital in India. Cureus.

[22] Kazemi E., Soldoozi Nejat R., Ashkan F., Sheibani H. (2021). The laboratory findings and different COVID-19 severities: A systematic review and meta-analysis. Ann. Clin. Microbiol. Antimicrob..

[23] Thompson J.V., Meghani N.J., Powell B.M., Newell I., Craven R., Skilton G., Bagg L.J., Yaqoob I., Dixon M.J., Evans E.J. (2020). Patient characteristics and predictors of mortality in 470 adults admitted to a district general hospital in England with COVID-19. Epidemiol. Infect..

[24] Hornick A., Tashtish N., Osnard M., Shah B., Bradigan A., Albar Z., Tomalka J., Dalton J., Sharma A., Sekaly R.P. (2020). Anisocytosis is Associated with Short-Term Mortality in COVID-19 and May Reflect Proinflammatory Signature in Uninfected Ambulatory Adults. Pathog. Immun..

